# Humoral response to anti-SARS-CoV-2 vaccine in breastfeeding mothers and mother-to-infant antibody transfer through breast milk

**DOI:** 10.1038/s41541-022-00499-5

**Published:** 2022-06-23

**Authors:** Carlo Pietrasanta, Abbass Darwich, Andrea Ronchi, Beatrice Crippa, Elena Spada, Fabio Mosca, Lorenza Pugni, Maria Rescigno

**Affiliations:** 1 Fondazione IRCCS Ca’ Granda Ospedale Maggiore Policlinico, NICU, Milan, Italy, Via della Commenda 12, 20122 Milan, Italy; 2 University of Milan, Department of Clinical Sciences and Community Health, Milan, Italy, Via Francesco Sforza 35, 20122 Milan, Italy; 3 IRCCS Humanitas Research Hospital, Rozzano Milan, Italy; 4 Department of Biomedical Sciences, Humanitas University, Pieve Emanuele Milan, Italy

**Keywords:** Paediatric research, Respiratory tract diseases

## Abstract

The magnitude of mother-to-infant transfer of anti-SARS-CoV-2 antibodies through breast milk (BM) after maternal vaccination during breastfeeding, in the absence of transplacental transfer of IgG, remains unclear. Here, we quantified anti-S and anti-RBD IgG, IgA, IgA1, and IgA2 in maternal serum, maternal saliva, BM, infant buccal swabs, and infant feces up to 90 days after the second maternal vaccine dose. BNT162b2 vaccine induced long-lasting IgG in maternal serum, but weaker mucosal antibody production, with anti-SARS-CoV-2 IgG and IgA amounts in BM between 10- and 150-fold lower compared to serum. BM IgA were exclusively of the IgA1 isotype, with no production of the mucosal-specific and protease-resistant IgA2. Accordingly, only traces of antibodies were retrieved from the feces of breastfed infants, and no IgG nor IgA were retrieved from infants’ buccal swabs. Newly engineered anti-SARS-CoV-2 vaccines may be needed to stimulate the antibody production at mucosal sites such as breast milk.

## Introduction

Early in life, infants benefit from passive humoral protection against several infectious diseases through the transplacental passage of maternal IgG antibodies during pregnancy, and through immunoglobulins transferred after birth with breast milk (BM)^1^. These antibodies may originate from natural maternal infection, or from maternal vaccinations administered either before, during or after pregnancy, such as that against influenza and pertussis^2^. Vaccinations administered during pregnancy can favor the passive protection of infants through the transplacental passage of specific antibodies, mainly IgG. Conversely, infants of mothers vaccinated after delivery can benefit exclusively from the mucosal transfer of specific antibodies through breast milk.

During the last 2 years, the COVID-19 pandemic has posed previously unmet challenges to healthcare providers of perinatal medicine: indeed, it is now clear that SARS-CoV-2 infection during pregnancy increases the risk of maternal mortality, intensive care admission and premature delivery, and that vertical transmission of SARS-CoV-2 to the fetus and the newborn occurs in a small percentage of cases^3–5^. SARS-CoV-2 infection during pregnancy elicits both systemic and mucosal immune response in the mothers, and the presence of antigen-specific IgG and IgA have been demonstrated in maternal serum, cord blood (in cases of infection during pregnancy), and BM^6–9^.

Even though pregnant and lactating women were excluded from clinical trials on COVID-19 vaccines for safety and ethical concerns, two mRNA vaccines were subsequently approved for use in both pregnant and lactating women^10^. Extensive observational research has demonstrated that mRNA vaccines are safe even in these categories, eliciting robust systemic humoral and cellular responses akin to those highlighted in non-pregnant or non-lactating women^11–13^. In adults, both COVID-19 mRNA vaccines seem to induce a weaker mucosal immune response compared to the systemic one, mirroring what is already known for several other vaccines administered systemically^14^. Few previous studies highlighted that some SARS-CoV-2-specific IgG, IgM and IgA, variably quantified as “titers” or absorbance in ELISA-based assays, can be recovered from the BM of vaccinated mothers^12,15,16^, but the lack of a consistent reference standard^17^ makes the precise quantification of antibodies in different biological specimens, and the comparison between studies, cumbersome.

Furthermore, the presence of maternally transmitted anti-SARS-CoV-2 antibodies on the mucosal surfaces of infants breastfed by vaccinated mothers has never been demonstrated, nor the clinical protection of breastfed infants possibly induced by the persistence of BM antibodies on mucosal surfaces, such as the upper respiratory tract or the gastrointestinal tract.

The objective of this study was to longitudinally evaluate the subclasses (IgG, IgA, IgA1, and IgA2) of anti-SARS-CoV-2 antibodies in maternal and neonatal samples after the administration of COVID-19 mRNA vaccine to breastfeeding mothers, quantifying them using the WHO International Standard for anti-SARS-CoV-2 immunoglobulin as reference.

## Results

### Study population

**Fig. 1:**
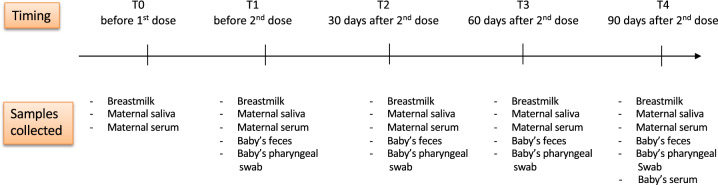
Study timeline. Timing of study visits and samples collection at each visit.

Women and infants provided biological samples at the time points indicated in Fig. 1. During the study period, 24 mothers and their singleton babies (13 males) were enrolled. One infant tested positive for anti-N antibodies, and his samples were excluded from data analysis. His mother repeatedly tested negative for anti-N antibodies. Demographic and clinical characteristics of the mother-infant pairs are reported in Table 1. Median maternal age at enrollment was 34 years (IQR: 33–39.5), and median infant age was 4.8 months (IQR: 2.6–7). Ninety-one percent (21/23) of neonates were term born, with a median GA of 39 weeks (IQR: 38–40). Most women (62.5%) were exclusively breastfeeding at T0 and T1, with a progressive reduction down to 13.6% at T4. In babies receiving partial breastfeeding, the median number of breast feedings per day was 4 (IQR: 2–10) at T0, 4 (IQR: 2–10) at T1, and 3 (IQR: 1–10) at T4. Side effects were reported by lactating mothers more frequently after the second vaccine dose (75%). Fifty-eight percent reported malaise, and 17% reported fever. None of the women reported a reduction in BM production following vaccination.

**Table 1:** Demographic and clinical characteristics of women and infants enrolled.

	No (%)	
No. of mothers	24	
No. of infants^a^	23	
Maternal age at 1st dose, median (IQR), y	34 (33–39)	
Mode of delivery		
Vaginal	19 (79)	
Exclusive breastfeeding at 1st dose	15 (65)	
Exclusive breastfeeding at 2nd dose 90 ± 1 days	3 (13)	
Gestational age, median (range), wks	39 (33–41)	
Preterm birth	2 (8)	
Birth weight, mean (SD), g	3455 (440)	
Infant age at 1st dose, median (IQR), wks	21 (11–30)	
	After 1st dose	After 2nd dose
Reported reaction	10 (41)	18 (75)
Injection-site pain	5 (21)	2 (8)
Malaise	1 (4)	14 (58)
Headache	1 (4)	7 (29)
Fever	0	4 (17)
Diarrhea	1 (4)	0
Myalgia	1 (4)	5 (21)
Nausea	2 (8)	2 (8)
Rash	0	1 (4)
Adenitis	0	1 (4)

^a^Samples from 1 neonate were excluded due to anti-N serum positivity at T3.

### Maternal systemic and mucosal humoral response

**Fig. 2:**
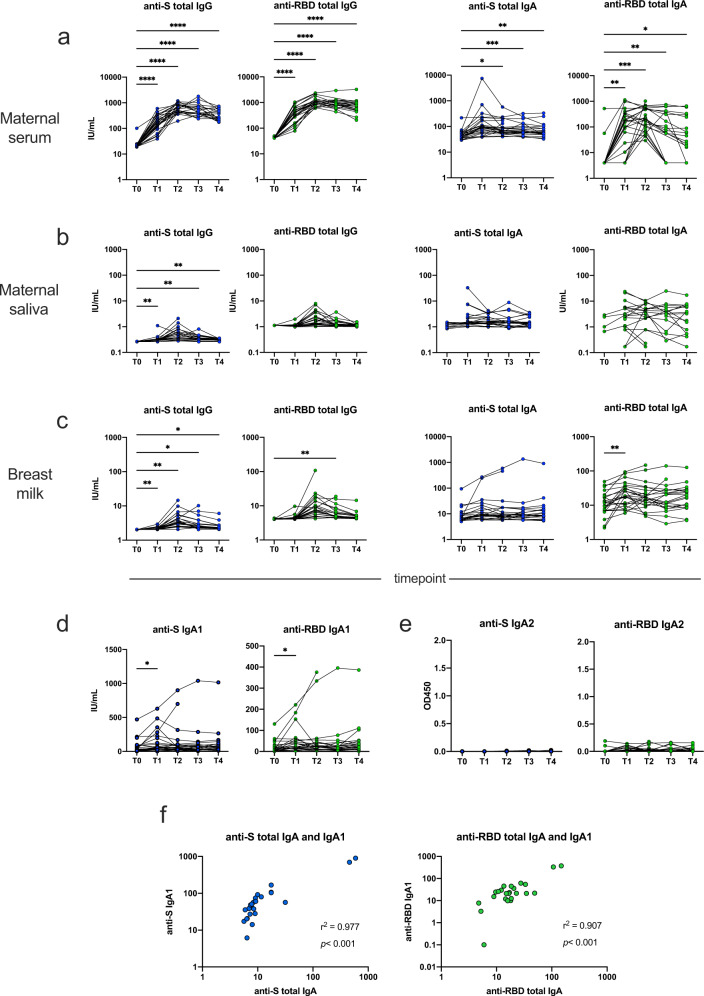
Antibody concentrations in maternal samples up to 90 days after receipt of BNT162b2 vaccine during lactation. **a**–**c** Antibody concentrations in maternal samples, after vaccination with BNT162b2 vaccine (*n* = 24). Subjects received the first dose at T0, and the second dose 21 days later, at T1. Then samples were collected every 30 ± 1 days until 90 ± 1 days after the second dose (T4). If there were no missing samples, *P* values were calculated by repeated-measures ANOVA with Dunnett’s correction for multiple comparison. In case of randomly missing samples, a mixed-effect model using the maximum likelihood method was applied. ****<0.001, ***<0.005, **<0.01, *<0.05. **d**, **e** titers of anti-SARS-CoV-2 IgA1 and IgA2 in breast milk at different timepoints. Statistic methodology as reported in (**a**–**c**). **f** Correlation between breast milk total anti-SARS-CoV-2 IgA and IgA1 concentration at 30 ± 1 days after vaccination (T2).

Following immunization, mothers mounted a robust systemic humoral response against both full-length S protein and its RBD, with a significant increase of serum IgG already after the first dose and stable concentrations until at least 90 days after the second dose (*p* < 0.001 for T1, T2, T3, and T4 vs. T0, Fig. 2a and Table 2). Concentrations peaked 30 days after the second dose, with a 26-fold increase compared to T0 (geometric mean: 581.9 IU/ml, 95% C.I: 486.5–696 vs. 22.4 IU/mL, 95% C.I. 19.5–25.7 for anti-S IgG, *p* < 0.001, Table 2). Anti-S serum IgA were 1.3- and 6.7-fold lower compared to IgG after the first and the second dose, respectively, while anti-RBD IgA significantly increased already after the first dose but were 4- and 6.8-fold lower that the corresponding IgG. RNA-based vaccine induced only a slight increase in salivary anti-S IgG compared to pre-vaccination status (Fig. 2b) and, interestingly, no significant anti-S or anti-RBD IgA response. When we investigated the pattern and kinetic of anti-SARS-CoV-2 antibodies in BM (Fig. 2c), we found similar trends: anti-S IgG 30 days after the second vaccine dose was 3.8 IU/mL (95% C.I.: 3.1–4.6), 153-fold lower compared to maternal serum. The concentration progressively declined until 90 days after the second dose, but remained significantly higher compared to pre-vaccination status and to the salivary one. BM anti-RBD IgG followed a similar kinetic while, conversely, both anti-S and anti-RBD IgA peaked earlier, already after the first dose, and then slightly decreased (Table 2, and Fig. 2c). Overall, we observed higher antibody concentrations in BM as compared to maternal saliva.

**Table 2:** Geometric means and 95% C.I. of antibody titers (IU/mL) in the maternal specimens analyzed^a^.

IgG geometric mean (95% C.I.)											
		T0		T1		T2		T3		T4	
Serum	Anti-S	22.37	(19.46–25.71)	160.6	(119.00–216.80)	581.9	(486.50–696)	507.2	(394.80–651.70)	357	(284.50–447.90)
	Anti-RBD	42.28	(41.49–43.08)	338.7	(253.50–452.40)	1089	(928.80–1278)	908.8	(752.20–1098)	703.3	(544.60–908.20)
Saliva	Anti S	0.26	(0.26–0.27)	0.33	(0.29–0.38)	0.5	(0.40–0.62)	0.37	(0.33–0.41)	0.32	(0.31–0.33)
	Anti-RBD	1.12	(1.12–1.12)	1.10	(1.03–1.17)	1.99	(1.53–2.57)	1.32	(1.14–1.53)	1.13	(1.08–1.18)
Breast milk	Anti-S	2.04	(2.03–2.05)	2.17	(2.10–2.25)	3.78	(3.11–4.60)	2.97	(2.47–3.56)	2.52	(2.26–2.82)
	Anti-RBD	4.06	(4.03–4.09)	4.42	(4.10–4.77)	9.19	(6.83–12.40)	6.21	(5.12–7.53)	5.02	(4.41–5.71)
IgA geometric mean (95% C.I.)											
		T0		T1		T2		T3		T4	
Serum	Aanti-S	45.2	(37.89–53.93)	119.1	(73.81–192.1)	87	(66.36–114.1)	81.09	(61.46–107)	69.54	(53.25–90.81)
	Anti-RBD	54.73	(34.22–87.52)	84.75	(39.22–183.1)	160.3	(94.25–272.7)	61.13	(23.89–156.4)	35.04	(14.57–84.31)
Saliva	Anti S	1.14	(0.973–1.33)	2.049	(1.40–3.01)	1.75	(1.47–2.08)	1.659	(1.27–2.17)	1.569	(1.28–1.92)
	Anti-RBD	0.23	(0.054–0.92)	1.69	(0.76–3.73)	1.43	(0.65–3.17)	0.92	(0.35–2.41)	0.91	(0.36–2.28)
Breast milk	Anti-S	8.54	(6.56–11.19)	13.37	(8.68–20.59)	12.88	(7.72–21.49)	11.95	(7.06–20.22)	12.87	(7.62–21.74)
	Anti-RBD	11.13	(7.97–15.54)	20.58	(14.76–28.68)	18.12	(12.78–25.68)	15.75	(10.54–23.53)	16.67	(11.25–24.7)

^a^No. of missing samples: 2 breast milks at T3, 2 breast milks at T4, 2 sera at T3, 2 sera at T4, 15 saliva at T0, 3 saliva at T1, 2 saliva at T3, 2 saliva at T4.

### Breast milk IgA subclasses

It is recognized that antibodies in mucosal secretions such as saliva and BM can result from both local production by activated mucosal plasma cells and from passive transfer of serum antibodies. Likewise, human IgA exist in 2 isoforms: IgA1, which is more abundant in the serum, and IgA2, which is found primarily in mucosal secretions and is more resistant to bacterial proteases, due to the lack of a 13-amino acid sequence containing a protease cleavage site in the hinge region^18^. To ascertain whether anti-SARS-CoV-2 IgA in BM were of systemic or mucosal origin, we separately quantified the amount of IgA1 and IgA2. As reported in Fig. 2d–f, BM IgA elicited by vaccination were almost exclusively of the IgA1 isoform. No IgA2 was retrievable from BM samples, while IgA1 titers correlated strongly with those of total IgA (*r*^*2*^ = 0.977 for anti-S, *r*^*2*^ = 0.907 for anti-RBD, all *p* < 0.001).

### Anti-SARS-CoV-2 antibodies on neonatal mucosal surfaces

**Fig. 3:**
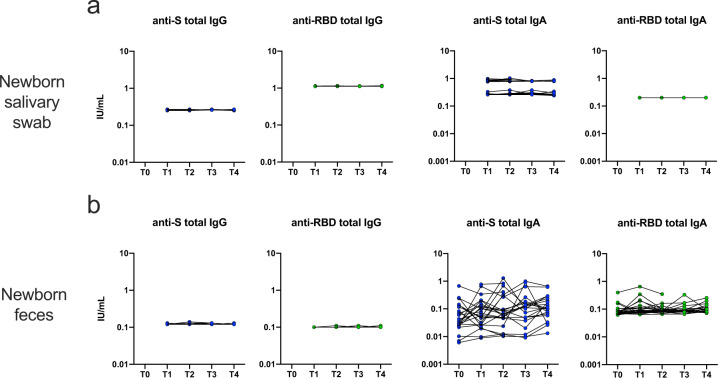
Antibody concentrations in neonatal mucosal samples up to 90 days after vaccination. **a**, **b** Antibody concentrations at different timepoints in neonatal samples, after maternal vaccination with BNT162b2 vaccine (*n* = 23). If there were no missing samples, *P* values were calculated by repeated-measures ANOVA with Dunnett’s correction for multiple comparison. In case of randomly missing samples, a mixed-effect model using the maximum likelihood method was applied.

It has been suggested that anti-SARS-Cov-2 antibodies in human milk could protect breastfed neonates against infection. However, considering the low absolute amount of both IgG and IgA produced in maternal mucosal fluids after vaccination, and the predominance of IgA1 isoform in BM, we wondered whether these antibodies could be recovered on neonatal mucosal surfaces, specifically on the buccal mucosa and in fecal samples. Using the same WHO reference standard, we quantified anti-S and anti-RBD antibodies of IgG and IgA classes in the supernatant of buccal swabs and in fecal pellets of breastfed neonates. As reported in Fig. 3a, we could not demonstrate the presence of significant amounts of anti-SARS-CoV-2 antibodies on neonatal buccal mucosa, neither IgGs nor IgAs. In neonatal feces (Fig. 3b) we could retrieve only limited amounts of anti-S and anti-RBD IgA, which however did not significantly increase over time (T2 geometric mean: 0.08 IU/mL, 95% C.I.: 0.05–0.15 for anti-S; 0.09 IU/mL, 95% C.I.: 0.08–0.1 for anti-RBD). This indicated that mother-derived antibodies were not preserved in neonatal mucosal secretions.

## Discussion

Vaccination has been universally recognized as the single most impactful weapon to overcome the effects of the COVID-19 pandemic^19^. In the context of perinatal medicine, the safety and effectiveness of anti-SARS-CoV-2 vaccination during pregnancy have been widely investigated: more than 100.000 pregnant vaccinated women are now recorded in the US-based v-safe registry^20^, and a significant clinical benefit, at least for the mothers, has been demonstrated in a growing number of observational cohorts^21,22^. Conversely, the immune response to maternal vaccination administered after delivery, during the lactation period, has only been marginally investigated, and the clinical benefits of the maternal antibody transfer through BM to the breastfed toddlers have only been hypothesized^23^.

Herein, we demonstrate that maternal immunization with BNT162b2 mRNA vaccine during the lactation period is safe, and effective in inducing a strong systemic humoral response in maternal serum, but it elicits a limited recovery of antibodies at mucosal sites. The small quantity of BM IgA detected following vaccination was almost entirely of the IgA1 subclass, with no protease-resistant IgA2. This suggests that IgA do not have a mucosal origin and is thus highly unstable. Accordingly, with the technical limits of our assay, we detected only minimal amounts of anti-SARS-CoV-2 antibodies in the feces of infants breastfed by vaccinated mothers. Our work presents three novelties in the field of maternal COVID-9 immunization. We are the first to evaluate antibody titers in mucosal samples of children breastfed by recently immunized mothers; we show that IgA are not of mucosal origin as they are all of the IgA1 subtype; we report absolute amounts of antibodies using the WHO International Standard, which has been recently indicated as the only internationally acceptable standard for the quantification of anti-SARS-CoV-2 antibodies in human specimens^17^. Our data on maternal serum confirmed the effectiveness of mRNA vaccine in inducing anti-SARS-CoV-2 IgG even when administered during lactation. Furthermore, IgG titers remained high for at least 90 days after the second dose, but further timepoints will be necessary to investigate the persistence of antibodies for longer periods. We also highlighted the different kinetic of serum IgG and IgA production after vaccination: indeed, IgA serum titer peaked after the first dose, and then declined, despite remaining higher than pre-vaccination status up to 90 days after the second dose. These data confirm those collected by others^12,16^. As regards antibodies in BM, already during the first year of pandemic it was demonstrated that SARS-CoV-2 natural infection elicited a mucosal humoral response both when infection was acquired during pregnancy and during lactation^7,8^. Antibodies recovered from BM were both IgG and IgA, and were mostly neutralizing^16^. The presence of anti-SARS-CoV-2 IgG and IgA in BM has also been reported after maternal vaccination administered both during pregnancy and lactation. The great difference between serum and BM titers that we found has been similarly reported by others^12,24^, despite it is impossible to directly compare the results because no previous study used the WHO international reference standard. Until now, indeed, published studies variably reported antibody levels in pregnant or lactating mothers as arbitrary unit (AU), optical density (OD) of ELISA-based assays, or “end point titer”, calculated over an assay-dependent threshold of detection, and with no concordance between different research groups^8,11–13,15,16^. The lack of a universal reference standard has complicated the interpretation of data as well as the comparison between studies, and has not allowed to identify a consistent correlate of protection. In December 2020, the first WHO International Standard for anti-SARS-CoV-2 immunoglobulin was established using freeze-dried pooled plasma obtained from subjects recovered from SARS-CoV-2 infection^25^. As such, the WHO Standard contains IgG, IgM and IgA against multiple SARS-CoV-2 antigens, including N protein, S protein, and RBD but, as we directly verified, no mucosa-specific IgA2 isotype. The WHO standard was made commercially available in early 2021. Therefore, we decided to take advantage of it in order to provide a universally accepted quantification of antibodies, and to directly compare the amounts of antibodies in different biological fluids.

The magnitude and kinetics of IgG and IgA responses that we highlighted in BM recalls that reported by Gray et al. and Perl et al.^12,15^, despite the methodological differences in the assays applied. After we highlighted such low BM IgA titers, and considered that several vaccines administered systemically are not capable of inducing significant antigen-specific mucosal responses^14^, we decided to quantify IgA1 and IgA2 isotypes. IgA1 are known to be predominant in serum, while IgA2 is the major subclass in secretions such as milk^18^. Moreover, IgA2 are induced by the presence of bacteria on mucosal surfaces^26^, are more resistant to bacterial proteases, and therefore are supposedly more likely to resist degradation on mucosal surfaces, such as the oral cavity, the upper respiratory trait, and the gastrointestinal lumen. We discovered that both anti-S and anti-RBD IgA2 are absent in BM after maternal vaccination, and that a strong correlation between total IgA and IgA1 isotype exists. Indeed, the presence of anti-SARS-CoV-2 IgA1 in BM, and the absence of IgA2, suggest that vaccine-induced antibodies are transferred to the BM from serum, rather than being produced by the activation of plasma cells in the mammary gland. Overall, our and previous data together corroborate the evidence that mRNA intramuscular anti-SARS-CoV-2 vaccination, akin to other systemically administered vaccines, elicits a significantly less robust mucosal than serological immune response in lactating mothers.

The presence of some vaccine-induced specific antibodies in BM led to the speculation that breastfed infants could benefit from some degree of passive protection through the transfer of antibodies to their mucosal surfaces, such as the upper respiratory airways and the gastrointestinal system^27^. Up to now, epidemiological evidence of this assumption is lacking, and considered the very low prevalence of symptomatic COVID-19 in lactating infants^28^, very large observational cohorts would be required to proof this hypothesis. We were not able to demonstrate significant amounts of antibodies in buccal swabs nor in the feces. We cannot exclude that more sensitive methods may retrieve some anti-SARS-CoV-2 antibodies, but the finding that IgA are of the IgA1 subtype suggests that they may not be detectable because of reduced resistance to enzymatic proteolysis. To the best of our knowledge, a limited amount of salivary/buccal mucosa antibodies has been recently demonstrated in breastfed infants after maternal infection, not after vaccination, and the presence of these immunoglobulin has been attributed by the authors to the direct activation of neonatal immune system against BM SARS-CoV-2 – Ig immune complexes^29^. Furthermore, only a pre-print, not peer reviewed report of fecal anti-SARS-CoV-2 antibodies recovered in breastfed neonates after maternal COVID-19 vaccination exists^30^: again, the authors did not report an absolute quantification of antibodies, nor a direct quantitative comparison between serum, milk, and fecal concentrations. Thus, it is virtually impossible to establish the real magnitude of this presumed antibody-transfer. We hypothesize that such small amounts of SARS-CoV-2 specific IgA1 and IgG, being more sensitive to bacterial proteases than IgA2, are easily cleaved in the non-sterile environment of the infant mouth, and for this reason, cannot be recovered in buccal swabs or from the lower gastrointestinal tract.

Our study presents some limitations. First, all women received BNT162b2 vaccine, as it was the only product recommended for the administration to lactating women in Italy during the enrollment. It is conceivable that mucosal responses after vaccination with other mRNA vaccines may be different, as recently hypothesized for the Moderna vaccine^31^. Second, we did not enroll a control group of infected lactating women, to directly compare antibody titers of vaccinated and infected women in different specimens. Third, we could not directly investigate the activation of mammary gland plasma cells following vaccination. Such evidence, either coming from human cohorts or animal models, may support a better comprehension of humoral mechanisms elicited by vaccination at the mucosal sites. Fourth, the inherent background of our IgA assay was higher than that of IgG: this technical bias has already been reported by others^16^, and could prove important for the detection of very small quantities of antibodies such as IgA in children’s samples. Finally, it’s important to note that antibody concentration is not always predictive of neutralizing activity, and that this can be retained by biological fluids even when small concentrations of antigen-specific antibodies are present^32^. Moreover, breast milk is a tissue rich in broadly-reacting antibodies, that might confer some degree of SARS-CoV-2 neutralization even in the absence of previous antigen exposure, as already demonstrated for other pathogens^33^.

In conclusion, in a cohort of lactating women receiving BNT162b2 vaccine and their breastfed infants we demonstrated a strong maternal systemic humoral response against SARS-CoV-2 Spike protein, but limited response at mucosal sites, specifically the mammary gland, with small amounts of antibodies found in BM and in the feces of breastfed infants. Our data point out the suboptimal stimulation of mucosal immune system by mRNA vaccines: the passive protection of infants breastfed by women vaccinated during lactation, in the absence of transplacental passage of antigen-specific IgG, is conceivable but yet to be proven.

## Methods

This was a prospective, single-center cohort study conducted at Fondazione IRCCS Ca’ Granda Ospedale Maggiore Policlinico of Milan, Italy, between May 23, 2021 and October 30, 2021.

### Study approval

The study protocol was approved by the Ethics Committee of the promoter center (Comitato Etico Milano Area B–Fondazione IRCCS Ca’ Granda Ospedale Maggiore Policlinico Milano, protocol number 2315) and the Ethics Committee of the Istituto Nazionale per le Malattie Infettive Lazzaro Spallanzani of Rome (appointed as National Ethics committee for the evaluation of clinical trials and medical devices for the treatment of patients affected by COVID-19. Approval number 267). Written informed consent was obtained from lactating mothers before they received the first dose of anti-SARS-CoV-2 vaccine, and all procedures were in accordance with the Declaration of Helsinki^34^.

### Study population

We recruited lactating women keen to receive anti-SARS-CoV-2 vaccine during breastfeeding, and determined to carry on breastfeeding for the following four months, either exclusive or partial with at least 50% of the total daily milk intake reported as breast milk (based on average intake per weight and self-reported amount of formula milk administered). All women received 2 doses of BNT162b2 anti-SARS-CoV-2 vaccine (Pfizer, Inc., and BioNTech) while breastfeeding, 21 days apart. Women and their infants provided biological samples at the following time points (Fig. 1): on the day of first vaccine dose (before administration, T0), on the day of the second vaccine dose (before administration, T1), and at 30 ± 1 (T2), 60 ± 1 (T3) and 90 ± 1 (T4) days after the second vaccine dose. Women reporting a proven previous episode of SARS-CoV-2 infection were excluded from enrollment, as well as those with a positive screening for anti-nucleocapsid (anti-N) antibodies at the time of enrollment. Furthermore, at every timepoint, maternal serological screening for anti-N antibodies was performed. Neonatal serological screening for anti-N was performed at T4, to exclude a previous neonatal infection. In case of neonatal anti-N positivity, neonatal specimens were excluded from the data analysis.

### Clinical data

The following maternal data were collected at enrollment and, where indicated, at each study time point: maternal age at vaccination, ethnicity, mode of delivery, type of breastfeeding at each timepoint (exclusive or partial), number of daily breast feeds at each timepoint. The following post-vaccination maternal symptoms were recorded: pain at the site of injection, malaise, fever >38 °C, headache, myalgia, nausea, skin rash, adenitis, diarrhea. Moreover, we recorded the following neonatal data: sex, gestational age (GA) at delivery, prematurity birth weight, age at maternal vaccination. Data from mothers and their neonates were prospectively collected using an electronic database.

### Samples collection

At every study visit, we collected maternal serum, maternal breast milk, maternal saliva, infant feces, and infant buccal mucosa swab. Infant blood for anti-N serological screening was collected only at T4. Maternal and infant blood was collected by venipuncture in serum separator tubes, and serum was obtained after centrifugation at 3000 × *g*. for 10 min at 4 °C. Sera were aliquoted in cryogenic vials and stored at −80 °C until further analysis. Breast milk was manually expressed by lactating mothers in the presence of a midwife, after nipple cleansing, and collected into study-provided containers. Milk was immediately aliquoted into multiple cryovials and frozen at −80 °C until analysis. Maternal saliva was collected fresh at the time of study visit, aliquoted, and frozen at −80 °C. Infant feces were collected by nursing mothers up to 24 h before the planned study visits into study-provided sterile containers, frozen at −20 °C, and subsequently transferred to −80 °C until further analysis. Infant swabs of the buccal mucosa were collected rotating gently a cotton swab (Copan Diagnostics) on the entire area of both left and right inner cheek, for 5 s each, and on the pharyngeal/tonsils area (5 s). The cotton swab was then transferred to a tube containing Universal Transfer Medium (UTM^®^) and frozen at −80 °C until analysis. Buccal swabs were collected a minimum of 2 h after the previous feeding, to avoid direct breast milk contamination.

### Samples preparation and antibodies detection

For the quantification of antibodies, serum, saliva, and breast milk were defrosted at room temperature (RT) and centrifuged for 5 min at 5000 × *g*. All samples were then diluted in Phosphate-buffered saline (PBS) with 1% human nonfat-dried milk (Sigma Aldrich). Serum was diluted 1:1000, breast milk (devoid of fat layer and cells) and maternal saliva 1:5. Buccal swabs were also defrosted at RT, then vortexed in their transport medium for 30 s, and the transport medium was diluted 1:2 in the same PBS 1% milk. Feces were defrosted at RT, suspended in PBS with 0.1 mg/ml of trypsin inhibitor from soybean (Sigma Aldrich) at a concentration of 100 mg/ml, and homogenized through shaking at 30 Hz for 20 s in a tissue lyser (Qiagen), after the addition of 1.4 mM ceramic beads (MP Biomedicals). They were then centrifuged at 50 × *g* for 10 min to remove debris, and supernatant was diluted 1:5 in PBS 1% milk.

Enzyme-linked immunosorbent assays (ELISAs) for the detection of anti-S, anti-RBD and anti-N IgG and IgA antibodies were performed on 96 wells flat bottom Nunc MaxiSorp plates, in technical duplicates. Briefly, each plate was coated with 2 μg/mL of full length Spike protein (S), Receptor Binding Domain (RBD), or Nucleocapsid (N) proteins (SinoBiological) from SARS-CoV-2 in PBS, and incubated at 4 °C overnight.

After incubation, plates were blotted dry and blocked with 300 μL of PBS with 3% human nonfat-dried milk for 1 h at RT. Then, block solution was discarded, and plates were blotted dry. Fifty μL of diluted samples were added to wells, and plates were incubated for 2 h at room temperature. Plates were then washed 3 times with wash buffer (0.1% Tween 20 in 1× PBS) and incubated at room temperature for 1 h with a 1:5000 dilution of mouse anti–human IgG horseradish peroxidase (HRP) conjugated (Genescript, clone 12H3C4A6 Catalog No. A01855) or a 1:10.000 dilution of goat anti–human IgA alkaline phosphatase (AP) conjugated (Thermo Fisher Scientific, RRID AB_2535561, Catalog No. A18784), or a 1:2000 dilution of mouse anti-human IgA1 AP conjugated (Southern Biotech, clone B3506B4, Catalog No. 9130-04), or a 1:8000 dilution of mouse anti-human IgA2 HRP conjugated (Southern Biotech, clone A9604D2, Catalog No. 9140-05). Plates were washed 3 times, and 50 μL of undiluted p-nitrophenyl phosphate (PNPP, Thermo Fisher Scientific) or 3,3′,5,5′-Tetramethylbenzidine (TMB) solution (Thermo Fisher Scientific) was added to each well; TMB reactions were stopped by adding 25 μL of 0.18 M sulfuric acid per well. The absorbance at 405 (PNPP) or 450 (TMB) nm was recorded with a ClarioStar (BMGLabtech) microplate reader. On each plate, two duplicates of a serial 1:3 dilution in PBS 1% milk of the World Health Organization (WHO) International Standard and Reference Panel for anti-SARS-CoV-2 antibody (NIBSC, UK) were used as standard curve, starting from a concentration of 30 IU/mL. Before quantifying antibody titers in complex biological matrices such as breast milk and fecal supernatants, we tested the performance of the anti-IgA and anti-IgG detection antibodies with WHO standard curves diluted in three negative (T0) samples of the matrices of interest, at the same dilution of tested samples, and compared with a standard curve diluted in PBS 1% milk. The correlations between optical density (OD) of the standard curve diluted in PBS 1% milk or in the different biological matrices are reported in Supplemental Fig. 1: *r*^*2*^ were consistently > 0.95, with no significant increase in background signal. To calculate antibody titers, absorbance (OD) values of each experimental sample were interpolated with the average standard curve after correction for the absorption of blank (1% milk in PBS) controls.

### Statistical analysis

Descriptive statistics were calculated using GraphPad Prism 9.0.1 (GraphPad Software) and Stata 13.0 (StataCorp LP). Continuous variables are presented as median with interquartile range (IQR), categorical variables as numbers and proportion. To ascertain differences in antibody concentration between different specimens at specific timepoints, t-test was applied. To ascertain longitudinal differences in antibody concentration, repeated-measures ANOVA was applied. In case of missing values, we analyzed the data by fitting a mixed that uses a compound symmetry covariance matrix and is fit using Restricted Maximum Likelihood. In the absence of missing values, this method gives the same *P* values and multiple comparisons tests as repeated measures ANOVA. In the presence of missing values (missing completely at random), the results can be interpreted like repeated measures ANOVA. Pearson’s *r* coefficient was used to assess the correlation between antibody concentrations. Reporting of results follows the Strengthening the Reporting of Observational Studies in Epidemiology reporting guidelines^35^.

### Reporting summary

Further information on research design is available in the Nature Research Reporting Summary linked to this article.

## Supplementary information

Supplemental material

Reporting Summary

## Data Availability

The complete study datasets are available to readers on reasonable request to the corresponding author.

## References

[1] Kollmann TR, Kampmann B, Mazmanian SK, Marchant A, Levy O (2017). Protecting the newborn and young infant from infectious diseases: Lessons from immune ontogeny. Immunity.

[2] Healy CM (2016). Pertussis vaccination in pregnancy. Hum. Vaccin. Immunother..

[3] Raschetti R (2020). Synthesis and systematic review of reported neonatal SARS-CoV-2 infections. Nat. Commun..

[4] Allotey J (2020). Clinical manifestations, risk factors, and maternal and perinatal outcomes of coronavirus disease 2019 in pregnancy: Living systematic review and meta-analysis. BMJ.

[5] Villar J (2021). Maternal and neonatal morbidity and mortality among pregnant women with and without COVID-19 infection: The INTERCOVID multinational cohort study. JAMA Pediatr..

[6] Ovies C, Semmes EC, Coyne CB (2021). Pregnancy influences immune responses to SARS-CoV-2. Sci. Transl. Med..

[7] Pace, R. M. et al. Characterization of SARS-CoV-2 RNA, antibodies, and neutralizing capacity in milk produced by women with COVID-19. *mBio*10.1128/mBio.03192-20 (2021). 10.1128/mBio.03192-20 PMC7885115 33563823

[8] Edlow AG (2020). Assessment of maternal and neonatal SARS-CoV-2 viral load, transplacental antibody transfer, and placental pathology in pregnancies during the COVID-19 pandemic. JAMA Netw. Open.

[9] Mithal LB, Otero S, Shanes ED, Goldstein JA, Miller ES (2021). Cord blood antibodies following maternal coronavirus disease 2019 vaccination during pregnancy. Am. J. Obstet. Gynecol..

[10] Walsh EE (2020). Safety and immunogenicity of two RNA-based Covid-19 vaccine candidates. N. Engl. J. Med..

[11] Collier AY (2021). Immunogenicity of COVID-19 mRNA vaccines in pregnant and lactating women. JAMA.

[12] Gray KJ (2021). Coronavirus disease 2019 vaccine response in pregnant and lactating women: A cohort study. Am. J. Obstet. Gynecol..

[13] Atyeo C (2021). COVID-19 mRNA vaccines drive differential antibody Fc-functional profiles in pregnant, lactating, and nonpregnant women. Sci. Transl. Med..

[14] Krammer F (2020). SARS-CoV-2 vaccines in development. Nature.

[15] Perl SH (2021). SARS-CoV-2-specific antibodies in breast milk after COVID-19 vaccination of breastfeeding women. JAMA.

[16] Young BE (2021). Association of human milk antibody induction, persistence, and neutralizing capacity with SARS-CoV-2 infection vs mRNA vaccination. JAMA Pediatr..

[17] Knezevic I (2021). WHO International Standard for evaluation of the antibody response to COVID-19 vaccines: call for urgent action by the scientific community. Lancet Microbe.

[18] Macpherson AJ, McCoy KD, Johansen FE, Brandtzaeg P (2008). The immune geography of IgA induction and function. Mucosal Immunol..

[19] Creech CB, Walker SC, Samuels RJ (2021). SARS-CoV-2 vaccines. JAMA.

[20] Moro, P. L., Panagiotakopoulos, L., Oduyebo, T., Olson, C. K. & Myers, T. Monitoring the safety of COVID-19 vaccines in pregnancy in the US. *Hum. Vaccin. Immunother.*10.1080/21645515.2021.1984132 (2021). 10.1080/21645515.2021.1984132 PMC8903939 34756131

[21] Dagan N (2021). Effectiveness of the BNT162b2 mRNA COVID-19 vaccine in pregnancy. Nat. Med..

[22] Goldshtein I (2021). Association between BNT162b2 vaccination and incidence of SARS-CoV-2 infection in pregnant women. JAMA.

[23] Hall S (2021). COVID vaccines and breastfeeding: What the data say. Nature.

[24] Charepe N (2021). COVID-19 mRNA vaccine and antibody response in lactating women: A prospective cohort study. BMC Pregnancy Childbirth.

[25] Kristiansen PA (2021). WHO International Standard for anti-SARS-CoV-2 immunoglobulin. Lancet.

[26] He B (2007). Intestinal bacteria trigger T cell-independent immunoglobulin A(2) class switching by inducing epithelial-cell secretion of the cytokine APRIL. Immunity.

[27] Jorgensen SC, Burry L, Tabbara N (2021). The role of maternal COVID-19 vaccination in providing immunological protection to the newborn. Pharmacotherapy.

[28] Pietrasanta C (2020). SARS-CoV-2 infection and neonates: A review of evidence and unresolved questions. Pediatr. Allergy Immunol..

[29] Conti MG (2021). Immune response of neonates born to mothers infected with SARS-CoV-2. JAMA Netw. Open.

[30] Narayanaswamy, V. et al. Breastfeeding infants receive neutralizing antibodies and cytokines from mothers immunized with a COVID-19 mRNA vaccine. *medRxiv*10.1101/2021.10.12.21264890 (2021).

[31] Yang, X. et al. Comparative Profiles of SARS-CoV-2 Spike-Specific Human Milk Antibodies Elicited by mRNA- and Adenovirus-Based COVID-19 Vaccines. *Breastfeed Med*. 10.1089/bfm.2022.0019 (2022). Online ahead of print. 10.1089/bfm.2022.0019 35675683

[32] Yeo KT (2021). Neutralizing activity and SARS-CoV-2 vaccine mRNA persistence in serum and breastmilk after BNT162b2 vaccination in lactating women. Front Immunol..

[33] Zheng W (2020). Microbiota-targeted maternal antibodies protect neonates from enteric infection. Nature.

[34] World Medical, A. (2013). World Medical Association Declaration of Helsinki: Ethical principles for medical research involving human subjects. JAMA.

[35] von Elm E (2007). Strengthening the Reporting of Observational Studies in Epidemiology (STROBE) statement: Guidelines for reporting observational studies. BMJ.

