# Supplementary material for: Humoral response to anti-SARS-CoV-2 vaccine in breastfeeding mothers and mother-to-infant antibody transfer through breast milk
Source: NPJ Vaccines. 2022 Jun 23;7:63. doi: 10.1038/s41541-022-00499-5 (PMC9226003; doi:10.1038/s41541-022-00499-5)
Supplement: Supplementary file 1 — Supplemental material [file 41541_2022_499_MOESM1_ESM.docx]

**SUPPLEMENTAL MATERIAL**

**Systemic and mucosal humoral response to anti-SARS-CoV-2 mRNA vaccine in breastfeeding mothers and mother-to-infant antibody transfer through breast milk**

Carlo Pietrasanta^1,2^, Abbass Darwich^3^, Andrea Ronchi^1^, Beatrice Crippa^1^, Elena Spada^1^, Fabio Mosca^1,2^, Lorenza Pugni^1^, Maria Rescigno^3^

^1^ Fondazione IRCCS Ca' Granda Ospedale Maggiore Policlinico, NICU, Milan, Italy. Via della Commenda 12, 20122 Milan, Italy

^2^ University of Milan. Department of Clinical Sciences and Community Health, Milan, Italy. Via Francesco Sforza 35, 20122 Milan, Italy

^3^ IRCCS Humanitas Research Hospital, Rozzano, Milan, Italy

^4^ Department of Biomedical Sciences, Humanitas University, Pieve Emanuele, Milan, Italy

**Corresponding Author**

Carlo Pietrasanta, MD, PhD

Department of Woman-Child-Newborn, Fondazione IRCCS Ca’ Granda Ospedale Maggiore Policlinico

Via Francesco Sforza 35, 20122 Milan, Italy

Phone: +39.5503.2907

Mail: carlo.pietrasanta@policlinico.mi.it

Mail: carlo.pietrasanta@gmail.com

**Supplemental Figure 1.** Correlations between anti-S and anti-RBD IgG and IgA titers of serial 1:3 dilutions of WHO International Standard for anti-SARS-CoV-2 immunoglobulin (standard curves) diluted in PBS 1% skim milk (blank) or in three negative samples of each complex biological matrix of samples analyzed in the study (saliva, breast milk, feces), diluted 1:5 in blank.
