# Supplementary material for: Humoral response to anti-SARS-CoV-2 vaccine in breastfeeding mothers and mother-to-infant antibody transfer through breast milk
Source: NPJ Vaccines. 2022 Jun 23;7:63. doi: 10.1038/s41541-022-00499-5 (PMC9226003; doi:10.1038/s41541-022-00499-5)
Supplement: Supplementary file 2 — Reporting Summary [file 41541_2022_499_MOESM2_ESM.pdf]

nature portfolio

Corresponding author(s): Carlo Pietrasanta

Last updated by author(s): Apr 11, 2022

Reporting Summary

Nature Portfolio wishes to improve the reproducibility of the work that we publish. This form provides structure for consistency and transparency in reporting. For further information on Nature Portfolio policies, see our Editorial Policies and the Editorial Policy Checklist.

Statistics

For all statistical analyses, confirm that the following items are present in the figure legend, table legend, main text, or Methods section.

n/a	Confirmed	
☐	☒	The exact sample size (*n*) for each experimental group/condition, given as a discrete number and unit of measurement
☐	☒	A statement on whether measurements were taken from distinct samples or whether the same sample was measured repeatedly
☐	☒	The statistical test(s) used AND whether they are one- or two-sided<br>*Only common tests should be described solely by name; describe more complex techniques in the Methods section.*
☒	☐	A description of all covariates tested
☐	☒	A description of any assumptions or corrections, such as tests of normality and adjustment for multiple comparisons
☐	☒	A full description of the statistical parameters including central tendency (e.g. means) or other basic estimates (e.g. regression coefficient) AND variation (e.g. standard deviation) or associated estimates of uncertainty (e.g. confidence intervals)
☐	☒	For null hypothesis testing, the test statistic (e.g. *F*, *t*, *r*) with confidence intervals, effect sizes, degrees of freedom and *P* value noted<br>*Give P values as exact values whenever suitable.*
☒	☐	For Bayesian analysis, information on the choice of priors and Markov chain Monte Carlo settings
☒	☐	For hierarchical and complex designs, identification of the appropriate level for tests and full reporting of outcomes
☐	☒	Estimates of effect sizes (e.g. Cohen's *d*, Pearson's *r*), indicating how they were calculated

Our web collection on statistics for biologists contains articles on many of the points above.

Software and code

Policy information about availability of computer code

Data collection	Microsoft Excel version 15.54, Microsoft corp
Data analysis	GraphPad Prism vers. 9.0.1, GraphPad Software LLC.

For manuscripts utilizing custom algorithms or software that are central to the research but not yet described in published literature, software must be made available to editors and reviewers. We strongly encourage code deposition in a community repository (e.g. GitHub). See the Nature Portfolio guidelines for submitting code & software for further information.

Data

Policy information about availability of data

All manuscripts must include a data availability statement. This statement should provide the following information, where applicable:

- Accession codes, unique identifiers, or web links for publicly available datasets
- A description of any restrictions on data availability
- For clinical datasets or third party data, please ensure that the statement adheres to our policy

complete database of de-identified data is available upon reasonable request to the corresponding Author

Field-specific reporting

Please select the one below that is the best fit for your research. If you are not sure, read the appropriate sections before making your selection.

☒ Life sciences ☐ Behavioural & social sciences ☐ Ecological, evolutionary & environmental sciences

For a reference copy of the document with all sections, see nature.com/documents/nr-reporting-summary-flat.pdf

Life sciences study design

All studies must disclose on these points even when the disclosure is negative.

Sample size	Sample size was not established a-priori. This is a cohort study where all available individuals over a pre-determined period of time were enrolled
Data exclusions	Only data from 1 neonate positive at anti-N screening were excluded from further analysis
Replication	This was a human study and data were not biologically replicated. However,assays were always run in technical duplicates
Randomization	cohort study with no randomization requested
Blinding	Investigators were not blinded to any treatment of enrolled patients

Reporting for specific materials, systems and methods

We require information from authors about some types of materials, experimental systems and methods used in many studies. Here, indicate whether each material, system or method listed is relevant to your study. If you are not sure if a list item applies to your research, read the appropriate section before selecting a response.

Materials & experimental systems

n/a	Involved in the study	
☐	☒	Antibodies
☒	☐	Eukaryotic cell lines
☒	☐	Palaeontology and archaeology
☒	☐	Animals and other organisms
☐	☒	Human research participants
☐	☒	Clinical data
☒	☐	Dual use research of concern

Methods

n/a	Involved in the study	
☒	☐	ChIP-seq
☒	☐	Flow cytometry
☒	☐	MRI-based neuroimaging

Antibodies

Antibodies used	- mouse anti–human IgG horseradish peroxidase (HRP) conjugated (Genescript), clone 12H3C4A6 Cat. No. A01855<br>- goat anti–human IgA alkaline phosphatase (AP) conjugated (Thermo Fisher Scientific), RRID AB_2535561, Catalog # A18784<br>- mouse anti-human IgA1 AP conjugated (Southern Biotech), B3506B4, Cat. No.: 9130-04<br>- mouse anti-human IgA2 HRP conjugated (Southern Biotech), A9604D2, Cat. No.: 9140-05
Validation	validation for each antibody used has been obtained by the manifacturer and is certified

Human research participants

Policy information about studies involving human research participants

Population characteristics	All involved patients were women of childbearing age and their neonates.Median maternal age at enrollment was 34 years (IQR: 33-39.5), and median infant age was 4.8 months (IQR: 2.6-7). Ninety- one percent (21/23) of neonates were term born, with a median GA of 39 weeks (IQR: 38-40). Most women (62.5%) were exclusively breastfeeding at T0 and T1, with a progressive reduction down to 13.6% at T4.
Recruitment	All but two enrolled women were employed at Fondazione IRCCS Ca' Granda Ospedale Maggiore Policlinico, Milan, Italy. However, they were not all healthcare providers, therefore there was no professional significant bias
Ethics oversight	The study protocol was approved by the Ethics Committee of the promoter center (Comitato Etico Milano Area B– Fondazione IRCCS Ca' Granda Ospedale Maggiore Policlinico Milano, protocol number 2315) and the Ethics Committee of the Istituto Nazionale per le Malattie Infettive Lazzaro Spallanzani of Rome (appointed as National Ethics committee for the evaluation of clinical trials and medical devices for the treatment of patients affected by COVID-19. Approval number 267).

Note that full information on the approval of the study protocol must also be provided in the manuscript.

Clinical data

Policy information about clinical studies
All manuscripts should comply with the ICMJE guidelines for publication of clinical research and a completed CONSORT checklist must be included with all submissions.

Clinical trial registration	this was a cohort study and was not recorded at clinicaltrials.gov
Study protocol	The full study protocol is accessible upon reasonable request to the corresponding Author
Data collection	between May 23, 2021 and October 30, 2021
Outcomes	pre-determined outcomes were antibody concentrations in biological fluids from mothers and their neonates, quantified using the WHO standard for anti-SARS-CoV-2 antibodies.
